# Elucidating the Role of the Mo_2_C/MgO Catalyst Interface in the Mechanism of the Reverse Water Gas Shift Reaction

**DOI:** 10.3390/nano15201591

**Published:** 2025-10-18

**Authors:** Cameron Holder, Andrew Shabaev, Jeffrey Baldwin, Heather Willauer

**Affiliations:** 1 US Naval Research Laboratory, Materials Science & Technology Division, Washington, DC 20375, USA; 2 US Naval Research Laboratory, Acoustics Division, Washington, DC 20375, USA

**Keywords:** reverse water gas shift, DFT, reaction mechanism, supported catalysts, molybdenum carbide, magnesium oxide

## Abstract

The reverse water gas shift reaction (RWGS) is a key step in the valorization of CO_2_ to value-added products such as fuel. Metal carbides, particularly molybdenum carbide (Mo_2_C), supported on transition metal oxide supports have been reported as promising materials to be used as catalysts for the low-temperature RWGS reaction. A deeper understanding of catalyst support interactions can be greatly beneficial for the development of better and more efficient catalysts in the future. To this end, this study computationally investigated the effect of the interaction between the Mo_2_C(001) surface and the MgO(001) surface on the RWGS mechanism. The RWGS mechanisms were explored at the Mo_2_C/MgO interface, as well as on the bare surface of Mo_2_C. While the pathway at the interface went through an associative-type mechanism and a carboxylate intermediate, the Mo_2_C surface was found to go through a redox-type mechanism. Interestingly, both the kinetics and thermodynamics of each pathway were similar, suggesting that the observed differences in the CO_2_ hydrogenation pathways were primarily limited by the diffusion of CO_2_ across the MgO surface rather than inhibitory energetics resulting from the interplay of the Mo_2_C material and MgO support.

## 1. Introduction

Recent efforts have been focused on developing sustainable pathways towards net-zero emissions through the efficient capture and valorization of CO_2_ into useful products ranging from commodities such as plastics or industrially relevant chemical feedstocks to the production of liquid hydrocarbon fuels [1,2,3,4,5]. In particular, the production of hydrocarbon fuel from readily available environmental sources presents a unique opportunity to generate fuels near or at the point of use, reducing logistical constraints involved with fuel transportation [6,7,8]. A well-established method to produce liquid hydrocarbons is the hydrogenation of CO_2_ through two thermochemical reactions, the reverse water gas shift (RWGS) and Fischer–Tropsch reactions [9]. The RWGS is critical in this process as it provides the necessary reactant, CO, required for the subsequent Fischer–Tropsch reaction. However, the RWGS is often hampered by its endothermicity, requiring elevated reaction temperatures for favorable product formation. These higher temperatures favor supported noble metals catalysts such as Pt or Pd where these materials show activity closer to the thermodynamic limit compared to their activity at lower temperatures [10,11]. However, these elevated temperatures can have adverse effects on the catalyst bed, which results in deactivation over short time periods in addition to constraining the reactor design [12,13,14]. Therefore, the development of materials that have increased activity, stability, and selectivity at lower reaction temperatures and understanding their associated reaction mechanisms are imperative for future generations of RWGS catalysts.

A class of materials that have been of interest as catalysts for the low-temperature RWGS reaction are transition metal carbides. In particular, carbides composed of molybdenum or tungsten have been heavily studied as they have activities that are similar to those of noble metals including Pt or Pd [12,15,16,17,18,19]. Often, these carbides are deposited onto high-surface-area supports that help to increase the particle dispersion as well as improve the catalytic stability [15,20,21]. The metal oxides that are often utilized as supports for the RWGS can be classified as irreducible or reducible supports. Irreducible supports are relatively benign and do not participate in the catalytic reaction; reducible supports have been shown to synergistically interact with the active catalytic species, undergoing a reduction and oxidation cycle that helps to further promote observed catalytic activity [22]. Previous work from our group has experimentally investigated the effects of reducible and irreducible metal oxides as supports for a Mo_2_C catalyst for the RWGS. We showed that the use of reducible metal oxides such as ceria resulted in the overreduction of the active Mo_2_C to an inactive Mo species, and as such, irreducible supports generally performed better when used as a support [15].

MgO is an irreducible metal oxide that has been demonstrated in various applications including catalysis, memory storage, and spintronics [23,24,25,26]. Due to its inherent basicity, MgO has been investigated as a potential material to be used as a CO_2_ adsorbent with applications such as adsorption–desorption cycles to assist in the purification of flue gas streams [27,28,29,30]. Despite its theoretical capabilities to uptake CO_2_, physical experiments examining the adsorption capacity of CO_2_ on MgO have demonstrated limited performance [28,29]. To this end, controlling the coordination environment of the lattice oxygen has been shown to have a large impact on the observed adsorption capacity. For instance, Jensen et al. and Karlsen et al. conducted computational density functional theory (DFT) studies on low index facets of MgO and demonstrated that sites with lower lattice oxygen coordination, such as those at steps or corner edges of the (001) surface, exhibited enhanced CO_2_ adsorption compared to terrace sites of the MgO(001) surface [31,32]. On a similar note, Lv et al. showed computationally that the introduction of point defects including dopants or vacancies significantly improved the adsorption energy on the MgO(001) surface [29]. Recent experimental efforts have expanded on this by developing a synthetic method to preferentially produce MgO nanoparticles with specific facets [33,34]. In particular, Mutch et al. demonstrated that the high concentration of low-coordinated sites increased the capability for CO_2_ capture, even after induced sintering at elevated temperatures [34]. The ongoing efforts to improve the ability for MgO to efficiently capture CO_2_, coupled with its chemical inertness and simple rock-salt crystal structure, makes it an ideal support material to computationally study in combination with the active Mo_2_C catalyst for the RWGS reaction.

Recent efforts to improve the catalytic activity of molybdenum carbide have focused on either reducing its dimensionality through the synthesis of an MXene phase or stabilizing small Mo_x_C particles on metal oxide supports [35,36,37,38,39]. In addition to theoretically favorable CO_2_ adsorption, MgO has been demonstrated as a support for catalysts including Ni, Ru, or Rh for the dry reformation of methane, which typically has reaction temperatures of 700–900 °C [40,41,42]. The utilization of MgO has been shown to stabilize various sizes of these metal nanoparticles, even at elevated temperatures which far exceed the temperatures that are anticipated for the low-temperature RWGS catalysts (300–400 °C) that are the focus of this work. Therefore, the marriage of MgO-supported Mo_2_C could represent a unique pathway towards stabilizing Mo_2_C particles of various sizes with a potential for increased CO_2_ adsorption.

It is generally accepted that the RWGS reaction occurs via two pathways, a redox mechanism or an associative mechanism [43,44]. In the redox mechanism, CO_2_ dissociates into carbon monoxide (CO*) and an adsorbed oxygen atom (O*), forming a surface oxidic species. The dissociation of H_2_ into separate H* atoms enables this oxide species to be further reduced into H_2_O [45]. On the other hand, the associative mechanism is mediated through the formation of either a carboxylate (COOH*) or formate (HCOO*) intermediate requiring that the H* atom interacts with the adsorbed CO_2_* molecule. These intermediates can be further hydrogenated to form the desired end products, CO and H_2_O [43,44,45]. For zero-valent metals including Pt, Pd, Cu or Ni, the dominant associative pathway can often be predicted by the H* atoms’ mobility on the surface. For noble metals which have high H* atom mobilities, the carboxylic acid pathway often dominates, whereas for non-noble metals that have lower H* atom mobilities, the formate pathway is more prevalent [46]. Prior experimental studies on α-Mo_2_C have indicated a similar trend, that the redox or formate pathways are more kinetically and thermodynamically favorable compared to the carboxylic acid pathway [47].

Herein, we describe efforts to elucidate the role of the Mo_2_C/MgO interface in stabilizing important intermediates to the RWGS through computational simulations. We propose that the mechanism through which CO_2_ is hydrogenated at the Mo_2_C/MgO interface varies compared to an unsupported Mo_2_C material. The differences in the observed mechanism between the two surfaces are proposed to arise from sterically confined CO_2_ when in the presence of H atoms that enable the activation and binding of CO_2_ to the Mo_2_C/MgO interface.

## 2. Materials and Methods

All DFT calculations in this study were conducted with the Vienna Ab-initio Simulation Package (VASP) [48]. The generalized gradient approximation (GGA) utilizing the Perdew–Burke–Ernzerhof (PBE) functional was employed to describe the electron exchange correlations. Furthermore, PBE projector augmented-wave (PAW) potentials were used to describe interactions between core and valence electrons [49,50]. Additionally, the DFT-D3(BJ) method was applied to include Van der Waals interaction corrections. All converged structures had an electronic energy cutoff threshold of 10^−6^ eV and an ionic force threshold of 0.02 eV Å^−1^. All calculations used the Monkhorst-Pack k-points mesh sampling, where the bulk slabs utilized a k-point grid of 3 × 3 × 1 and the Mo_2_C/MgO structure used a k-point grid of 1 × 3 × 1 [51]. The climbing image nudged elastic band (CINEB) method was used for transition state calculations and to determine the minimum energy pathways [52]. Each reaction step included 3 intermediate images in between the chosen initial and final images.

For β-Mo_2_C(001), the unit cell was orthorhombic and had dimensions of 4.743 × 6.058 × 5.231 Å. This unit cell was composed of 4 C and 8 Mo atoms equating to 12 total atoms. The Mo_2_C(010) bulk slab was arranged in a (2 × 2 × 2) supercell with dimensions of 9.486 × 12.116 × 10.461 Å and a total of 96 atoms. The vacuum space above the unit cell in the z-direction was set at 35.922 Å. The MgO(001) had a cubic unit cell with dimensions of 4.238 × 4.238 × 4.238 Å. For the bulk MgO slab, a (3 × 3 × 3) supercell was constructed with dimensions of 12.714 × 12.714 × 32.714 Å and a total of 216 atoms.

Starting from the developed MgO(001) and Mo_2_C(001) surfaces described above, slight adjustment were made to the bulk supercells in order to simulate a simple model of Mo_2_C(010) deposited onto MgO(001). For a basis, the MgO support, was modelled using a (1 × 7 × 3) supercell after which a small island of Mo_2_C was placed atop at one end of the y-direction with the Mo_2_C(010) facet being in contact with the MgO(001) surface. The Mo_2_C island was modelled by a (1 × 2 × 2) supercell of Mo_2_C being continuous in the x-direction. To promote interaction with the MgO(001) surface, one layer of C atoms (4 in total) was removed at the interface between the Mo_2_C island the MgO support. This brought the number of atoms in the Mo_2_C island from 48 total atoms down to 44 total atoms. The Mo_2_C(001) facet was the facet pointed in the direction of the extended MgO support. The final structure had dimensions of 5.994 × 20.978 × 62.714 Å where the z-direction had excess room for a vacuum gap and the total number of atoms was 212 atoms (84 Mg, 84 O, 12 C, and 32 Mo).

The calculated molecular adsorption energies are given by Equation (1):

(1) $$E_{ads}=\;E_{molecule/substrate}-E_{molecule}-E_{substrate}$$

where *E_ads_* is the calculated adsorption energy, *E_molecule/substrate_* is the relaxed energy of the adsorbate interacting with the slab or substrate, *E_molecule_* is the energy of the molecule in the gas phase, and *E_substrate_* is the energy of the slab or substrate. All transition state energies as well as energies along the minimum energy pathway are given relative to the initial image of the mechanistic pathway. For both associative mechanisms, this image would be adsorbed (CO_2_ + H), while for the redox pathway, this would be an adsorbed CO_2_.

## 3. Results

It can be challenging to accurately model and computationally study a complex structure such as the Mo_2_C/MgO interface. As described in Section 2, the relaxed ensemble had had large supercell dimensions of 5.994 × 20.978 × 62.714 Å in addition to a large total number of atoms, numbering 212. Due to both its large unit cell and total number of atoms, numerous approximations were required in order to effectively model this interface. With respect to the following discussion, which examined various adsorbates on multiple surfaces, one important approximation to note was dispersion interactions such as van der Waals forces, which were addressed through the utilization of the DFT-D3 method. In particular, the DFT-D3(BJ) package was used to accommodate for the anticipated shorter atomic distances of the adsorbates on the surface [53].

### 3.1. Adsorption of Key Molecules on MgO(001) and Mo_2_C(001)

**Figure 1:**
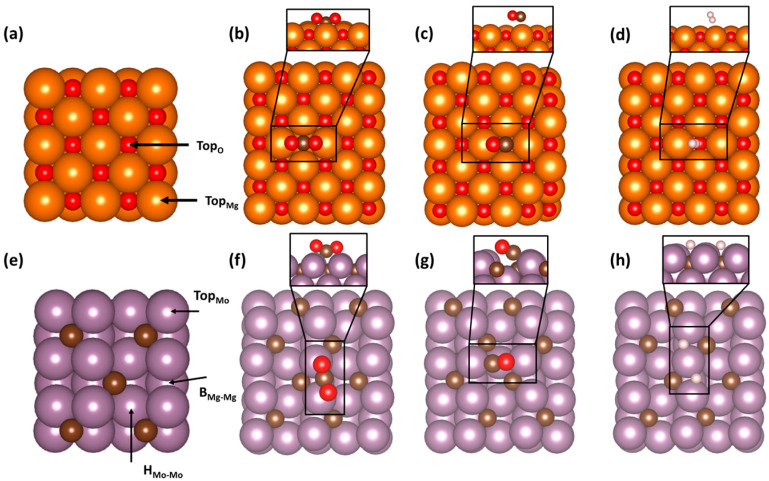
(**a**,**e**) Starting slabs of MgO(001) and Mo_2_C(001) with labelled adsorption sites, respectively. Relaxed positions of different adsorbates including (**b**,**f**) CO_2_, (**c**,**g**) CO, and (**d**,**h**) H_2_ adsorbed onto (**top row**) MgO(001) or (**bottom row**) Mo_2_C(001). Atoms of Mg, O, C, H, and Mo are indicated as orange, red, brown, white, and purple spheres, respectively.

Adsorbate interactions were first modelled on the bare surfaces of MgO(001) and Mo_2_C(001) as shown in Figure 1. For the MgO surface, two different adsorption sites, T_Mg_ and T_O_, were explored, and the most stable position was found to be the T_O_ position (Figure 1a). CO_2_ interacts favorably with the MgO(001) surface, adopting a bent structure with an O-C-O angle of 133° (Figure 1b). Additionally, the activated CO_2_ molecule had C-O bond distances that were elongated by approximately 7.2%. While CO_2_ showed favorable interactions with the MgO surface, both CO and H_2_ weakly interacted with the surface (Figure 1c and d, respectively). Instead, both molecules remained detached from the surface regardless of the T_O_ or T_Mg_ starting position, with H_2_ and CO being found approximately 2.5 Å to 2.9 Å above the MgO surface, respectively. The increases to the H-H and C-O bond lengths were nominal, indicating that any interaction with the nearby MgO surface was minimal.

Three unique sites on the Mo_2_C(001) were probed, the top of a Mo atom (T_Mo_), the hollow between adjacent Mo atoms (H_Mo-Mo_), and a bridge between Mo atoms (B_Mo-Mo_). These sites are displayed in Figure 1e. Upon relaxation, all three adsorbates appeared to strongly interact with the surface, albeit at different sites with the most stable configurations for each adsorbate being shown in Figure 1f–h. The CO_2_ relaxed into a bent configuration, preferentially adsorbing on the B_Mo-Mo_ site. The O-C-O angle of the CO_2_ molecule was 132° and the C=O bonds elongated by 8.7% compared to a standard gas-phase CO_2_ molecule. This configuration had each oxygen in proximity to a surface Mo atom with the carbon pointed towards the surface. Furthermore, when CO was allowed to relax, it was found to favorably bind at the H_Mo-Mo_ site. Unlike with CO_2_, the CO molecule was adsorbed to the surface primarily through the carbon atom. As similarly observed with CO_2_, the C-O bond distance for CO also increased by approximately 0.1 Å compared to an isolated CO molecule in vacuum. Finally, when H_2_ was placed onto the Mo_2_C(001) surface, it dissociated into separate H atoms on neighboring B_Mo-Mo_ sites. The distance between the two H atoms was 3.0 Å, which was significantly greater than a typical H-H bond length of 0.74 Å.

Table 1 lists the calculated adsorption energies of the adsorbates on each surface. The energies of the gas-phase molecules and the slab energies of both Mo_2_C(001) and MgO(001) are shown in Table S1. The adsorption energies for CO_2_, CO, and H_2_ on the Mo_2_C(001) surface were all <−1 eV, which indicated strong interactions with the surface. The CO_2_ and CO molecules showed the strongest adsorption, with energies of −2.37 and −2.23 eV, respectively. The adsorbed H_2_ molecule had an adsorption energy of −1.93 eV. As mentioned above, the H_2_ molecule was found to be dissociated on the Mo_2_C surface, and therefore, the energy of single H atom would be half of the energy for H_2_ tabulated in Table 1, or −0.97 eV. The adsorption values shown in Table 1 for the Mo_2_C surface aligns with what has been previously reported in the literature, though small differences are attributed to the difference in the utilized pseudo-potentials. For the MgO(001) surface, the reported energies are significantly more positive than those calculated for the Mo_2_C surface, indicating a preference for CO_2_, CO, and H_2_ to interact with the Mo_2_C surface compared to the MgO surface. This result was not surprising as Mo_2_C is, on its own, an active RWGS catalyst, and it is anticipated that the surface would be amenable to interactions with these adsorbates. CO_2_ had the strongest adsorption with an energy of −0.70 eV, while both CO and H_2_ showed little affinity towards interactions with the surface with adsorption energies of −0.17 and −0.12 eV, respectively. These results correlate well with the images presented in Figure 1.

### 3.2. Adsorption of Key Molecules at the Mo_2_C/MgO Interface

The molecular adsorption energies for CO_2_, H_2_, and CO when placed at the interface of a Mo_2_C ribbon atop a MgO(001) slab were determined utilizing methods similar to the studies above. The differences in adsorption energy as well as the reaction mechanisms between the Mo_2_C/MgO substrate and the bare surfaces of Mo_2_C(001) and MgO(001) were investigated and are further discussed below.

**Figure 2:**
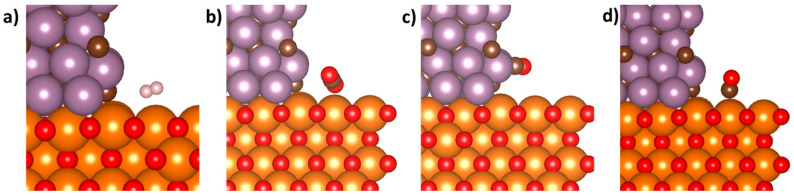
Relaxed images of (**a**) H_2_, (**b**) CO_2_ and (**c**,**d**) CO at two different positions after placement at the interface of a Mo_2_C ribbon atop a MgO substrate.

For both Mo_2_C and MgO, a molecule of H_2_ was placed on the surface and allowed to relax. As discussed previously, the H_2_ dissociated on the surface of Mo_2_C into 2 individually adsorbed H atoms. However, on MgO(001), the H_2_ molecule did not appear to dissociate and instead remained as a singular molecule that showed minimal interaction with the surface (Figure 2a). Interestingly, when placed near the Mo_2_C/MgO interface, the H_2_ molecule behaves similarly to what was observed on the MgO surface. Initially, H_2_ was positioned such that it was approximately 2 Å from either the MgO or Mo_2_C slabs and the H-H bond distance was 0.73 Å. This 2 Å distance from either the Mo_2_C ribbon or MgO slab mirrored the experiments conducted on the bare surfaces. After relaxation, the H_2_ molecule did not dissociate and had moved further away from the interface with the H-H bond length a nominal increase from its original position. From this result, individual H atoms were then investigated under the working hypothesis that hydrogen spillover from the Mo_2_C surface could supply the interface with the H atoms needed for the RWGS. This hydrogen spillover effect has been readily demonstrated for a multitude of different thermochemical catalysts [54,55,56]. When individual H atoms were placed on the surface of the ribbon, they readily adsorbed to both the MgO slab and the Mo_2_C ribbon (Figure S1). This indicated that the migration of individual H atoms through hydrogen spillover had the possibility to find an energy minimum that enabled adsorption on the surface of MgO or near the Mo_2_C/MgO interface, whereas the formation of these species through a H_2_ dissociation mechanism appeared to be unfavorable. From the results above on the bare surfaces, the Mo_2_C is anticipated to be supplying the interface with the H atoms needed to sustain catalytic activity for the RWGS.

Next, CO_2_ was placed near the Mo_2_C/MgO interface. The CO_2_ molecule was positioned such that it was initially parallel to the Mo_2_C ribbon. The molecule was placed 2 Å above the MgO and away from the Mo_2_C. The C-O bond distances before relaxation were 1.16 Å. After relaxation, the CO_2_ molecule did not show any interaction with the interface or either surface (Figure 2b). Instead, the CO_2_ molecule had rotated such that one oxygen was pointed towards the MgO support. The CO_2_ remained nearly linear with the O-C-O bond angle slightly bending to 177°; however, the O-C-O bond angle that was observed here was significantly more linear than compared to the Mo_2_C surface where the O-C-O bond angle was observed to be 132°. The C-O bond distances increased by 1.7% from a linear CO_2_ molecule. Since the interface appeared to weakly activate the CO_2_ molecule, it is unsurprising that the calculated adsorption energy of the CO_2_ molecule in this configuration was only −0.47 eV, indicating a weak interaction at the Mo_2_C/MgO ribbon interface.

Finally, CO was placed near the interface in two different starting configurations and allowed to relax. In both initial positions, the CO was oriented parallel to the Mo_2_C ribbon, and the carbon atom was placed on top of an oxygen site on the MgO slab (T_O_). The oxygen was then placed on either side of this carbon atom, resulting in the two different starting positions. The CO molecule in both initial starting positions was 2.5 Å away from the Mo_2_C and 2 Å above the MgO slab. Interestingly, the minor differences in starting position appeared to heavily influence the favored adsorption site as depicted in Figure 2c,d, respectively. Surprisingly, no favorable CO adsorption sites were found directly at the interface after relaxation. Instead, the CO molecule was found on either the Mo_2_C or MgO surfaces. It was energetically more favorable for the CO to interact with the Mo_2_C ribbon than the underlying MgO slab by 1.70 eV. The interaction between the CO and the Mo_2_C(001) correlates well with the CO interactions observed on the bare Mo_2_C(001) slab that was discussed earlier. Additionally, the interaction between the CO molecule and the MgO support was 0.3 eV stronger when compared to the bare MgO itself, which indicated some mutually beneficial electronic effect of the nearby Mo_2_C ribbon.

### 3.3. Investigating the RWGS Pathway on Mo_2_C and Mo_2_C/MgO

As discussed above, the two primary reaction pathways for the RWGS are the associative and redox pathways, with the associate pathway going through an intermediate species of formate (HCOO*) or carboxylate (COOH*). It should also be noted that while the following discussion is focused on the two primary pathways for the reduction of CO_2_ to CO in an ideal vacuum, practical systems will have varied kinetics and thermodynamics due to various factors that are not considered in this study. These factors can include but are not limited to undesired side reactions like the Sabatier reaction, variations in temperature and gaseous pressure throughout the catalyst bed, as well as catalyst deactivation.

#### 3.3.1. Adsorption of CO_2_ and an Individual H Atom at the Mo_2_C/MgO Interface

**Figure 3:**
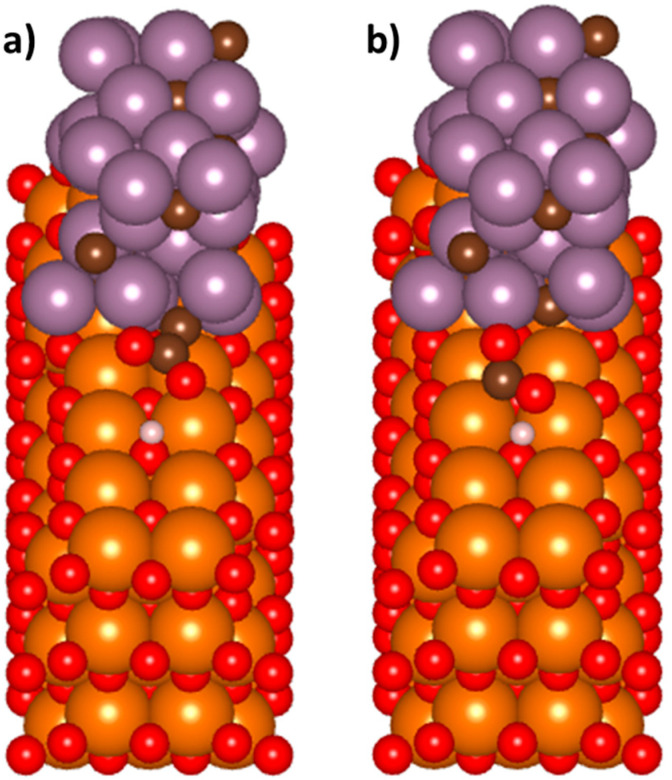
(**a**) An activated CO_2_ molecule parallel to the Mo_2_C/MgO interface with a nearby H atom and (**b**) an activated CO_2_ molecule perpendicular to the Mo_2_C/MgO interface with a nearby H atom.

The weak interaction of CO_2_ at the Mo_2_C/MgO interface presented in Figure 2 and tabulated in Table 1 indicated that a redox mechanism that involved an adsorbed CO_2_ dissociating into a CO* and O* as well as the dissociation of H_2_ into separate H atoms would be highly unlikely. Computational efforts were therefore focused on determining the energy barriers related to the associative mechanism and the two different intermediate species. To begin, CO_2_* and H* were modelled on the surface of the ribbon. The H was placed on the MgO support on a T_O_ site, which had been previously shown to be an energetic minimum. The CO_2_ was placed similar to that described above, parallel to both the Mo_2_C ribbon and the MgO support and approximately 2 Å away from each surface. There was 2.83 Å separating the CO_2_ molecule from the H atom in the initial setup. Interestingly, and in contrast to what was observed for the interactions of a lone CO_2_ molecule with the Mo_2_C/MgO ribbon, the presence of a nearby H* atom caused the CO_2_* molecule to interact with the ribbon at the interface, adopting a bent configuration as shown in Figure 3a. The C-O bond lengths increased by 9.4% and 12.8%, respectively, while the O-C-O bond angle decreased from 180° to 117.8°, an even more activated species than what was observed on the bare surfaces of Mo_2_C or MgO. However, if the H atom was placed on a site that is slightly further away from the Mo_2_C ribbon, the CO_2_ molecule does not interact with the Mo_2_C/MgO. Instead, a structure like that discussed previously for a lone CO_2_ molecule is observed (Figure S2). Combining these results with the observations that were discussed previously, the H* spillover from the dissociation of H_2_ on the surface of the Mo_2_C is crucial to the activation of the CO_2_ at the interface.

The orientation of the CO_2_ at the Mo_2_C/MgO interface was found to be an important factor in determining the favorable RWGS mechanism. For instance, the CO_2_ orientation shown in Figure 3a indicates that the carbon atom is relatively inaccessible to an attack from a nearby H atom. Instead, the formation of a COOH* intermediate is more plausible since it would not require a large steric re-arrangement. We briefly examined a different CO_2_ orientation that would enable access to the carbon atom which would be imperative in the formation of a formate intermediate, HCOO*. As shown in Figure 3b, the CO_2_* was anchored to both the Mo_2_C and MgO slabs through the oxygens of the CO_2_* molecule. Nearby, a H* atom is in a similar position as observed for the other CO_2_* configuration depicted in Figure 3a. The relaxed image demonstrated that the C=O bond distances increased by 10.8% and 12.1%, with the most elongated bond being the one closest to the nearby H*. The O=C=O bond angle was similar to that observed for the configuration in Figure 3a at 115.8°. After compensating for the substrate, the relaxed energy of the image containing the bidentate interaction of the CO_2_* and Mo_2_C slab (Figure 3a) was found to be −1.48 eV, while the energy of the image with the bidentate CO_2_* and MgO slab (Figure 3b) interaction was −0.38 eV. Therefore, the CO_2_* anchored to the MgO with the more accessible carbon is 1.10 eV less favorable than the CO_2_* anchored to the ribbon. While we cannot rule out the possibility that there exists other CO_2_ configurations that have more favorable energies, the results presented here suggested that the most stable system at the interface of the Mo_2_C/MgO slab comprised a CO_2_ molecule that had accessible oxygen atoms versus an accessible carbon atom. The orientation and associated energies of the activated CO_2_ molecules at the Mo_2_C/MgO interface indicated that the COOH pathway may be more thermodynamically favorable compared to the HCOO pathway.

#### 3.3.2. Associative Mechanism (*COOH) at the Mo_2_C/MgO Interface

To examine this hypothesis, an associative pathway going through a carboxylate intermediate was first investigated. The climbing image nudged elastic band (CINEB) method was used to determine transition states and energy barriers for the proposed reaction mechanism. Since the adsorption process of the CO_2_ molecule and H atom has been previously discussed, we started with an initial state comprising an adsorbed CO_2_ and H near the Mo_2_C/MgO interface. Literature reports have indicated that a bound COOH* molecule will dissociate into a COH* and O* before further dissociation leads to a system containing two unique species, CO* and OH* [47,57]. In order to generate this intermediate image, we relaxed a slab with an adsorbed COH* and O* where the C-O bond to the lone oxygen was broken and this oxygen was then moved towards the MgO surface. Upon relaxation, instead of observing the anticipated O* and COH* molecules, the hydrogen of the COH* was found to transfer to the nearby lone O*. This stable geometry resulted in an image that contained a CO* and OH* molecule. Therefore, a mechanistic step that involved a direct pathway towards the dissociation of the carboxylate intermediate, starting from COOH* and resulting with CO* + OH*, was hypothesized.

**Figure 4:**
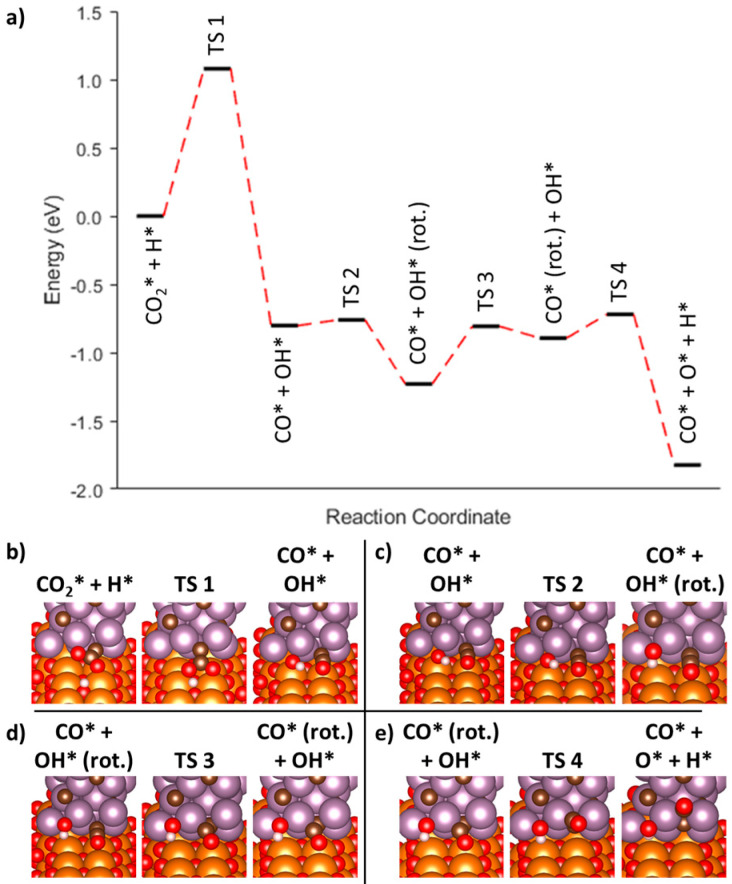
(**a**) Minimum energy pathway for the associative mechanism through a carboxylate intermediate at the Mo_2_C/MgO interface. (**b**–**e**) Representative images of the initial, transition state, and final configurations for the adsorbates for each step along the mechanism.

To test the validity of this statement, the minimum energy pathway was calculated for the associative mechanism through a carboxylate intermediate and is presented in Figure 4a. Additionally, the images of the initial, transition, and final states for each reaction step are also presented Figure 4b–e. The first initial image started with CO_2_ and H on the ribbon’s surface. Efforts to converge on a final image that contained a carboxylate intermediate instead resulted in separate CO and OH species. Upon connecting the initial CO_2_ + H image and the final CO + OH image through three intermediate images, the pathway appeared to go through a COOH* transition state (TS 1, Figure 4b), with the barrier being calculated at 1.08 eV (AC1-1, Table 2). The resulting CO* and OH* intermediate had a final energy of −0.80 eV. Efforts to get to the final CO* + O* + H* geometry from the CO* + OH* intermediate state were surprisingly unsuccessful due to the presence of local energy minima along this pathway which hindered complete relaxation. To accommodate for these minima, both the CO* and OH* moieties were rotated in subsequent steps to enable the further dissociation of the OH* into separate O* and H* atoms (AC1-2 and AC1-3, Table 2). The rotation of the OH molecule resulted in an CO* + OH* (rot.) image with a calculated energy of −1.23 eV and an associated reaction barrier of 0.04 eV (Figure 4c). Further rotation of the adsorbed CO* was slightly less energetically favorable with the resulting CO* (rot.) + OH* having an energy of −0.89 eV and a transition barrier of 0.43 eV (Figure 4d). A final barrier with a height of 0.17 eV resulted in the desired formation of the CO* + O* + H* final image with an energy of −1.82 eV relative to the initial CO_2_* and H* adsorbates (Figure 4e). The final image consisting of three different adsorbates, CO* + O* + H*, was 0.93 eV lower in energy than the preceding CO* + OH* image, suggesting that the former image with three adsorbates is more thermodynamically stable at the interface than just having CO* + OH* (AC2, Table 2). The overall process described herein is slightly different than the conventional pathway described in the literature. There does not appear to be an energetically stable configuration where COH* + O* is stable at the Mo_2_C/MgO interface. Instead, adsorbed CO* + OH* can result directly from the hydrogenation of CO_2_ and an H atom which can then be transformed into the final three adsorbate configuration of CO* + O* + H*. This pathway is outlined in Table 2.

#### 3.3.3. Associative Mechanism (*HCOO) at the Mo_2_C/MgO Interface

**Figure 5:**
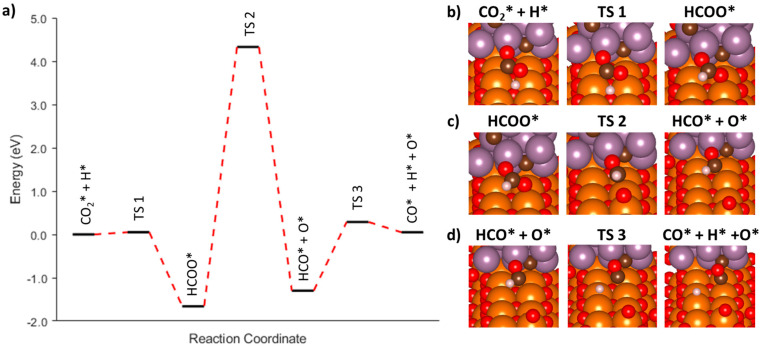
(**a**) Minimum energy pathway for the associative mechanism through a formate intermediate at the Mo_2_C/MgO interface. (**b**–**d**) Representative initial, intermediate, and final images for each reaction step.

Though the associative mechanism through a formate intermediate was anticipated to be less favorable due to the converged energy of the CO_2_* + H* depicted in Figure 3b, efforts using the CINEB method were also done to examine this pathway at the Mo_2_C/MgO interface. Similar to the investigation of the associative carboxylate mechanism above, the minimum energy pathway in addition to the initial, intermediate, and final images for the key steps in the associative formate mechanism is shown in Figure 5. The initial step along this pathway started with the relaxed image presented in Figure 5b and ended with HCOO*. The adsorbed HCOO* had a calculated adsorption energy of −1.66 eV with a small energy barrier of 0.05 eV implying a kinetically fast step (AF1-1, Table 2). The next mechanistic step resulted in the dissociation of the HCOO* into HCO* + O*. This step appeared to be severely limited kinetically due to a calculated energy barrier of 5.99 eV. Meanwhile, the HCO* + O* image had a final calculated energy of −1.30 eV (AF2, Table 2). The final step in the process was the deprotonation of the HCO* to form three separate products, CO* + O* + H*. The final CO* + O* + H* configuration had a calculated energy of 0.05 eV, slightly higher than the CO_2_ + H starting image (AF3, Table 2). The energy barrier of the final step was 1.57 eV, which was significantly less energy intensive than the previous step. The large kinetic barrier for AF2 would represent a significant challenge to overcome, and its anticipated that this pathway is much less favorable than the carboxylate pathway described previously.

#### 3.3.4. Redox Mechanism on the Mo_2_C(001) Surface

In order to compare the energies discussed above pertaining to the RWGS at the Mo_2_C/MgO interface, we also explored various RWGS mechanisms over bare Mo_2_C as it is also known as an active RWGS catalyst. Bare MgO was excluded from these studies because it is not considered to be active for the RWGS and the results above indicated that there was no meaningful dissociation of H_2_ on its surface, a crucial process that is required for the RWGS reaction to progress. Unlike the interface, both CO_2_ and H_2_ had strong interactions with the Mo_2_C surface, and therefore, both the redox and associative mechanisms were investigated. Previous reports have suggested that the redox pathway is often the most favorable on the surface of Mo_2_C; however, the formate associative pathway has also been demonstrated [47].

**Figure 6:**
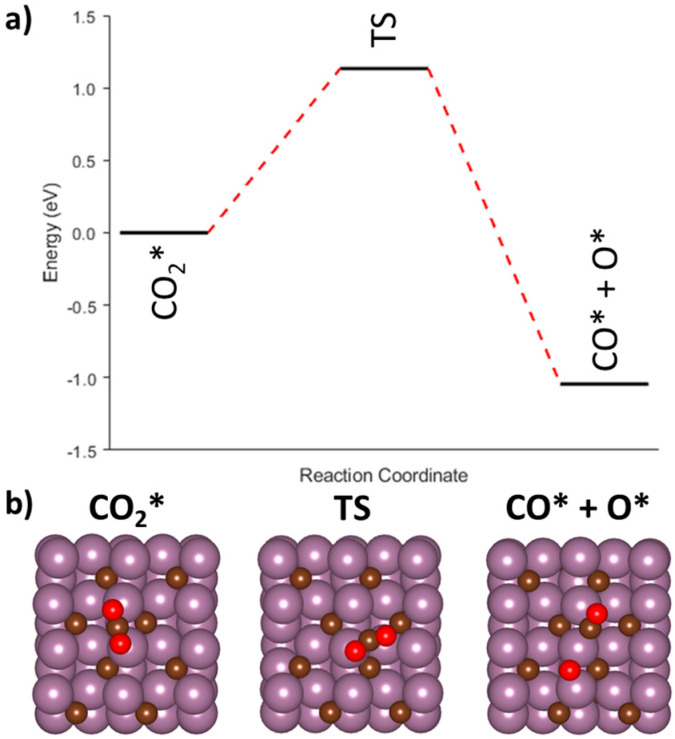
(**a**) Minimum energy pathway for the redox mechanism on Mo_2_C(001). (**b**) Representative initial, intermediate, and final images for dissociation of CO_2_ into CO and O.

We first investigated the redox mechanism over Mo_2_C(001). Initially, the CO_2_ is adsorbed at the B_Mo-Mo_ site where it is found to be activated with a corresponding O-C-O bond angle of 132.1°. The direct dissociation of CO_2_ to CO* + O* was investigated utilizing the CINEB method (R1, Table 2). The overall process was thermodynamically favorable with a ΔE of −1.04 eV, and the calculated transition barrier was 1.13 eV (Figure 6a). The CO* and O* moieties were spatially separated in the final image by roughly 3.19 Å (Figure 6b), and further studies were undertaken to determine if they were electronically separated. Both CO* and O* were individually modelled and relaxed with energies of −17.01 eV and −9.28 eV, respectively, after adjusting for the Mo_2_C slab energy. If the CO* and O* were electronically separated, it was anticipated that the summation of the individual energies of CO* and O* would be approximately equal to the model of both CO* and O* on the surface. The summation of the individual models equated to −26.29 eV, while the combined CO* and O* had a calculated energy of −25.57 eV, which represented a difference of −0.72 eV. Since there was a sizeable difference between the two energies, it was concluded that, while the O* and CO* were spatially separated from one another, the two adsorbates were still within close enough proximity to experience close by electronic perturbations. These electronic interactions could have an impact on their diffusion across the Mo_2_C surface or the desired desorption of CO.

#### 3.3.5. Associative Mechanism (HCOO) on the Mo_2_C(001) Surface

**Figure 7:**
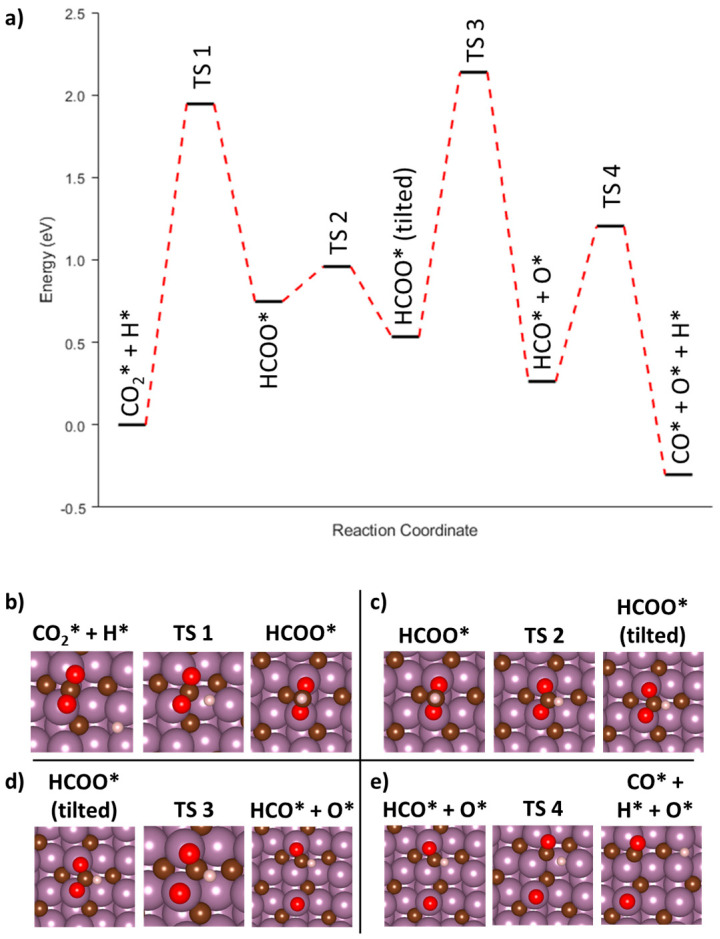
(**a**) Minimum energy pathway for the associative mechanism on Mo_2_C(001) going through a formate intermediate. (**b**–**e**) Representative initial, intermediate, and final images for each reaction step.

In addition to the redox mechanism, we also explored both the formate and carboxylate associative pathways. When examining the carboxylate pathway on the surface of Mo_2_C, it became apparent that a minimum energy pathway from the initial CO_2_* + H* to the desired reaction intermediate, COOH*, was hampered by large, non-converging forces. Any attempts to determine if other stable configurations existed along this pathway were unsuccessful, and therefore, efforts were refocused on the associative formate pathway. The minimum energy pathway and its associated images are shown in Figure 7. The first energetic barrier was determined to be 1.94 eV starting from an initial state of adsorbed CO_2_* + H* and ending in the formation of a formate (HCOO*) molecule (Figure 7b). The HCOO* had an adsorption energy of −2.83 eV, which was approximately 0.75 eV uphill from the initial starting configuration (AF1-1, Table 2). The reason for the large energy penalty for the formation of HCOO* in this instance was anticipated to be due to the starting configuration of the CO_2_ where, sterically, the carbon atom was beneath the two oxygen atoms to start. However, during the formation of the HCOO* intermediate, the CO_2_ molecule appeared to rotate such that the final configuration of the AF1 step resulted in the oxygens interacting with the Mo_2_C surface rather than the carbon atom. The minimum energy pathway for initial attempts at modelling the AF1-2 reaction step resulted in the formation of an intermediate image, which demonstrated that the HCOO* molecule would rotate such that the H-C bond would be interacting with the surface of the Mo_2_C (Figure 7c). The rotation of the HCOO adsorbate came at a minimal energy penalty of 0.21 eV. The tilted HCOO* had a final adsorption energy of −3.04 eV, which was 0.21 eV more favorable than the upright configuration. Upon maximizing the interaction of the HCOO* with Mo_2_C surface, the AF2 reaction step from Table 2 was successfully modelled. The barrier for this transition was 1.02 eV, while the ending adsorbates of HCO* + O* had a final energy of −0.27 eV, indicating the dissociation of the HCOO* is thermodynamically favorable though it may be kinetically limited. The final reaction step AF3 is shown in Figure 7e, which depicts the further dissociation of HCO* into CO* + H* as well as the residual O* from the previous step. Similar to prior steps in this mechanistic pathway, the AF3 reaction step appears to be downhill with the final image having a normalized energy of −0.56 eV. However, the energy barrier for AF3 is relatively large at 0.94 eV. Overall, the entire process starting from the two adsorbed species of CO_2_* + H* to the final configuration of CO* + O* + H* is downhill, while the intermediate reaction step AF1-1 appeared to be the rate-limiting step with a large barrier of 1.95 eV.

## 4. Discussion

As demonstrated above, the Mo_2_C/MgO interface and the bare surface of Mo_2_C appear to go through two different pathways for the RWGS reaction, the former being through a carboxylate-type intermediate and the latter preferring a redox-type mechanism. Addressing the bare surface of Mo_2_C first, Porosoff et al. showed spectroscopically that the surface of Mo_2_C could undergo oxidation under operating conditions to form a molybdenum oxycarbide [19,58]. This species was hypothesized as the true active material and that the RWGS reaction hinged on its formation. The results presented here on the Mo_2_C(001) surface aligns well with this experimental study, showing a feasible pathway of producing CO_2_ directly from its dissociation through the oxidation of the Mo_2_C surface. Additionally, the Mo_2_C can readily dissociate H_2_ into separate H atoms, which can then be used to reduce the oxycarbide species, forming water, and closing the catalytic cycle. Experimental investigations undertaken by our group for Mo_2_C supported on MgO showed poor catalytic performance, drastically underperforming when compared to Mo_2_C supported on γ-Al_2_O_3_ [15]. However, the computational results presented here indicated that the kinetic reaction barriers through a carboxylate intermediate were not all too different than those observed for a redox-type mechanism on the Mo_2_C surface, where the rate-limiting step in the favored associative mechanism for Mo_2_C/MgO was approximately 1.08 eV, while the respective rate-limiting step for the redox mechanism on Mo_2_C was 1.13 eV. Additionally, the Mo_2_C/MgO surface had more favorable thermodynamics through the carboxylate intermediate, being −1.82 eV downhill vs. −1.04 eV for the redox mechanism on Mo_2_C. These DFT results suggested that the poor experimental performance observed for the Mo_2_C/MgO system may not be due to the coupling of Mo_2_C/MgO but rather to the insufficient flux or diffusion of the CO_2_ reactant to the interface.

Our results herein demonstrated that CO_2_ is weakly physisorbed onto the surface of MgO(001) and the dissociation of H_2_ at this surface does not occur. The inability to form separate H atoms concurs with the knowledge that MgO is inactive for the RWGS. It then follows that any reaction occurring at the interface would require the diffusion of CO_2_ to the interface or the direct adsorption of CO_2_ at the interface from the gas phase. Taking previously experimental data regarding surface area and particle sizes as a basis for the calculations presented in the Supplementary Materials, the collision rate of CO_2_ from the gas phase was found to be roughly an order of magnitude less at the Mo_2_C/MgO interface than compared to the exposed surfaces of Mo_2_C or MgO [15]. Therefore, the primary method to obtain CO_2_ at the Mo_2_C/MgO interface would be surface diffusion. Additionally, the surface area of an oxide support often contributes much more significantly to a material’s total surface area than the catalytically active species. It can therefore be hypothesized that due to the larger proportionate surface area of the MgO support, and hence larger amount of CO_2_ collisions from the gas phase, as well as the fact that MgO does not participate in the RWGS reaction, there would exist a large CO_2_ concentration gradient between the interface and the MgO surface, which may be leveraged in future synthetic studies to help facilitate diffusion. The diffusion of CO_2_ on the MgO(001) surface was investigated by Meixner et al., and their findings showed that there was no detectable diffusion of CO_2_ along the MgO(001) surface at 100 K, with the upper limit of the diffusion coefficient being assigned at 1 × 10^−9^ cm^2^ s^−1^ [59]. Our brief investigations mirrored these findings where a minimum energy pathway for the diffusion of CO_2_ along either the x- or y-direction atop the MgO(001) could not be determined. Therefore, the inability for the adsorbed CO_2_ to diffuse across the support surface to the catalytically active sites at the interface would severely hinder the RWGS activity and thus could be a rational explanation to the experimental results previously reported. Future efforts to help increase both the diffusion and adsorption of CO_2_ on the MgO support may include the incorporation of oxygen vacancies or dopants such as Li, Na, or K, as well as the evaluation of various facets of MgO [27,34,60,61,62]. Additionally, any developed pathway should also consider its ability to scale towards industrial processes for future integration. These efforts represent intriguing pathways toward next-generation Mo_2_C/MgO catalysts that could improve upon the baseline RWGS activity.

## 5. Conclusions

Various mechanisms of the reverse water gas shift over Mo_2_C supported on MgO were investigated to determine differences in preferred reaction pathways compared to bare Mo_2_C. Initial studies looked at the adsorption energies of different molecules, and, importantly, H_2_ dissociation was found to occur on the bare Mo_2_C(001) surface. This dissociation is important due to hydrogen spillover to the Mo_2_C/MgO interface, where the inclusion of a H atom assists in the activation and binding of CO_2_ enabling the associative mechanism through a carboxylate intermediate. Comparatively, the reaction mechanism on a bare Mo_2_C surface indicated that the preferred pathway is either a redox pathway or an associative pathway through a formate intermediate. The difference in the preferred RWGS mechanism between the two surfaces is most likely due to the lower energy of the sterically confined CO_2_ molecule at the Mo_2_C/MgO interface, which likely directs the formation of the COOH intermediate. This study highlights the importance of understanding the impact that various oxide supports have on the RWGS pathway and will be essential in the development of future experimental catalysts with enhanced RWGS kinetics.

## Figures and Tables

**Table 1:** Calculated adsorption energies for various adsorbates on bare surfaces of MgO(001) and Mo_2_C(001), as well as Mo_2_C/MgO.

Molecule and Surface	Adsorption Energy (eV)
CO @ Mo_2_C(001)	−2.23
H_2_ @ Mo_2_C(001)	−1.93
CO_2_ @ Mo_2_C(001)	−2.37
CO @ MgO(001)	−0.17
H_2_ @ MgO(001)	−0.12
CO_2_ @ MgO(001)	−0.70
CO_2_ @ Mo_2_C/MgO	−0.47
H @ Mo_2_C/MgO	−0.57
CO @ Mo_2_C/MgO	−2.16

**Table 2:** Tabulated thermodynamic reaction energies (E_React_) and activation energies (E_act_) for the RWGS reaction mechanisms at the Mo_2_C/MgO interface and the Mo_2_C(001) surface.

Step	Reaction	Mo_2_C/MgO		Mo_2_C(001)	
		E_React_ (eV)	E_act_ (eV)	E_React_ (eV)	E_act_ (eV)
**Associative Carboxylate Mechanism**					
AC1-1	CO_2_* + H* → CO* + OH*	−0.80	1.08	---	---
AC1-2	CO* + OH* → CO* + OH* (rot.)	−0.43	0.04	---	---
AC1-3	CO* + OH* (rot.) → CO* (rot.) + OH*	0.34	0.42	---	---
AC2	CO* (rot.) + OH* → CO* + O* + H*	−0.93	0.17	---	---
	**Associative Formate Mechanism**				
AF1-1	CO_2_* + H* → HCOO*	−1.66	0.05	0.75	1.95
AF1-2	HCOO* → HCOO* (tilt)	---	---	−0.21	0.21
AF2	HCOO* → HCO* + O*	−1.30	5.99	−0.27	1.02
AF3	HCO* + O* → CO* + O* + H*	0.05	1.57	−0.56	0.94
	**Redox Mechanism**				
R1	CO_2_* → CO* + O*	---	---	−1.04	1.13

## Data Availability

The raw data supporting the conclusions of this article will be made available by the authors on request.

## Supplementary Materials

The following supporting information can be downloaded at: https://www.mdpi.com/article/10.3390/nano15201591/s1, Table S1: Energy of adsorbates including CO_2_, CO, and H_2_ as well as the bare slabs of MgO(001), Mo_2_C(001), and Mo_2_C/MgO. Figure S1. (a,c) Individual H atom adsorbed at the Mo_2_C/MgO interface. (b,d) Individual H atom adsorbed on the MgO support, away from the Mo_2_C/MgO interface. Figure S2. Relaxed images from two different angles, demonstrating that the proximity of an individual H atom to the Mo_2_C ribbon is important to activate CO_2_.

## Abbreviations

The following abbreviations are used in this manuscript:

DFT	Density Theory Functional
RWGS	Reverse Water Gas Shift
FT	Fischer–Tropsch Reaction
CINEB	Climbing Image Nudged Elastic Band
