# Supplementary material for: Elucidating the Role of the Mo2C/MgO Catalyst Interface in the Mechanism of the Reverse Water Gas Shift Reaction
Source: Nanomaterials (Basel). 2025 Oct 18;15(20):1591. doi: 10.3390/nano15201591 (PMC12567052; doi:10.3390/nano15201591)
Supplement: Supplementary file 1 [file nanomaterials-15-01591-s001.zip › nanomaterials-3913220-supplementary.pdf]

SUPPORTING INFORMATION

Elucidating the Role of the Mo_2C/MgO Catalyst Interface in the Mechanism of the Reverse Water Gas Shift Reaction

Cameron Holder[1*], Andrew Shabaev[1], Jeffrey Baldwin[2], Heather Willauer[1]

[1] US Naval Research Laboratory, Materials Science & Technology Division, Washington, DC 20375, USA

[2] US Naval Research Laboratory, Acoustics Division, Washington, DC 20375, USA

* Corresponding *Email Address: cameron.f.holder.civ@us.navy.mil*

Rate of CO_2 collisions with different surfaces calculation:

The flux of gaseous molecules interacting with the catalyst surface was determined through the Hertz-Knudsen equation (equation S1):

$$\frac{dN}{A \times dt} = \frac{P}{\sqrt{2 \times \pi \times m \times k_B \times T}} \qquad \text{(S1)}$$

where A is the area, P is the partial pressure of gas, m is the mass of a gaseous molecule, k_b is Boltzmann's constant, and T is temperature. The area of the catalyst surface, partial pressures of gas, and the reaction temperature were taken from our previously reported results (cite).

The area of the MgO substrate was determined by using the surface area determined by the Braunner-Emmett-Teller (BET) method, 29.6 m^2/g, reported previously.[1] Since 80% of the 0.35 g catalyst was composed of MgO, this meant that the MgO had a total surface area of 82,880 cm^2 (Equation S2).

$$29.6\ \frac{m^2}{g} \times 0.8 \times 0.35g \times \frac{10000\ cm^2}{m^2} = 82{,}880\ cm^2 \qquad (S2)$$

Using this area and knowing that the mass of a CO_2 molecule (7.31×10^{-26} kg), equation 1 can be solved for the amount of collisions per given time at the surface of MgO (Equation S3).

$$\frac{dN}{dt} = \frac{P\ \times A}{\sqrt{2 \times \pi \times m \times k_B \times T}}$$

$$\frac{dN}{dt} = \frac{403{,}000\ Pa\ \times 8.288\ m^2}{\sqrt{2 \times \pi \times (7.31 \times 10^{-26} kg) \times \left(1.3806\ \times\ 10^{-23}\ \frac{m^2 \times kg}{s^2 \times K}\right) \times 573\ K}} \qquad \text{(S3)}$$

$$\frac{dN}{dt} = \ 5.54\ \times 10^{28}\ \frac{collisions}{second}$$

Therefore, the amount of collisions of CO_2 at the MgO surface of a typical RWGS catalyst was predicted to be 5.54×10^{28} collisions per second, assuming a catalyst loading of 20%, a reactor pressure of 403 kPa, and a reactor temperature of 300 °C or 573 K.

A similar calculation can be completed for the Mo_2C particles as well as the Mo_2C/MgO interface, assuming that the interface is approximately 1 nm or 10 Å thick. The surface area of the Mo_2C particles was calculated to be 2.90 m^2 while the surface area of the total interfacial region was estimated to be 0.161 m^2. The interfacial region calculation is shown below in through the following equations.

The average Mo_2C particle diameter as determined by the Scherrer analysis in our previous report was 25 nm on the surface of MgO.[1] The volume of any given Mo_2C particle is then given by (Eqn. S4):

$$V = \frac{2}{3}\pi r^3 = \frac{2}{3} \times \pi \times 12.5\ nm^3 = 4090\ nm^3 \qquad \text{(S4)}$$

Where r is the radius of the particle. Using r = 12.5 nm, the total volume equates to 4090 nm^3. Using the density of a Mo_2C particle which is 9.18 g cm^{-3}, the mass per particle can be determined (Eqn. S5):

$$\frac{4.09 \times 10^{-18}\, cm^3}{particle} \times \frac{9.18\, g}{cm^3} = 3.76 \times 10^{-17} \frac{g}{particle} \quad (S5)$$

The total weight percentage of Mo on the experimentally tested catalyst was 20 wt% and the total catalyst weight was 0.35 g. This equates to 0.07 g of Mo or 0.074 g Mo_2C. Taking the result from equation 5, it can be calculated that there is approximately 1.97 x 10^{15} Mo_2C particles with an average radius of 12.5 nm supported on MgO. To calculate the annulus of a ring around a single Mo_2C particle assuming that the ring is 1 nm thick can be determined as follows (Eqn. S6):

$$Annulus = \pi \times (R^2 - r^2) \quad (S6)$$

Where R is the radius of the outer circle (Mo_2C particle radius and the 1 nm thick ring) and r is the radius of the inner circle (Mo_2C particle radius). Filling in and solving resulted in:

$$Annulus = \pi \times ([13.5\, nm]^2 - [12.5\, nm]^2) = 81.7\, nm^2 = 8.17 \times 10^{-17} m^2$$

From above, the total number of Mo_2C particles had already been determined, 1.97 x 10^{15} Mo_2C particles. Therefore, the total area of a 1 nm thick interface around all Mo_2C particles is then found to be the number given above, 0.161 m^2.

Using these two values, A_{Mo2C} and $A_{interface}$ as 2.90 and 0.16 m^2, respectively, the collisions of CO_2 on each surface as given by equation 1 can be determined. The collisions of CO_2 on the surface of Mo_2C is thus 1.94 x 10^{28} collisions per second while at the 1 nm interface, it is 1.08 x 10^{27} collisions per second.

Table S1: Energy of adsorbates including CO_2, CO, and H_2 as well as the bare slabs of MgO(001), Mo_2C(001), and Mo_2C/MgO

**Molecule and Surface**	Energy (eV)
CO_2 molecule	-22.95
CO molecule	-14.78
H_2 molecule	-6.77
MgO(001) Slab	-1309.32
Mo_2C(001) Slab	-981.43
Mo_2C/MgO Substrate	-1460.66

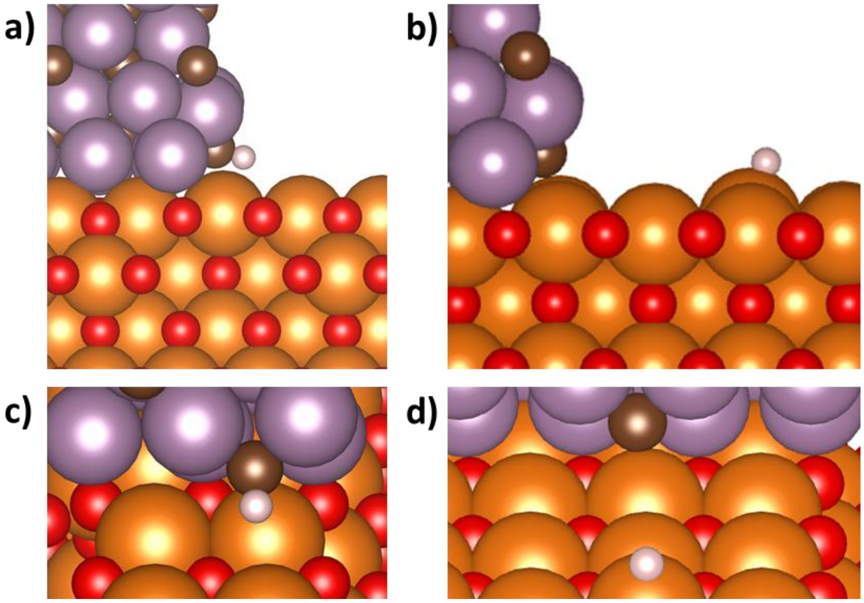

Figure S1. (a,c) Individual H atom adsorbed at the Mo_2C/MgO interface. (b,d) Individual H atom adsorbed on the MgO support, away from the Mo_2C/MgO interface.

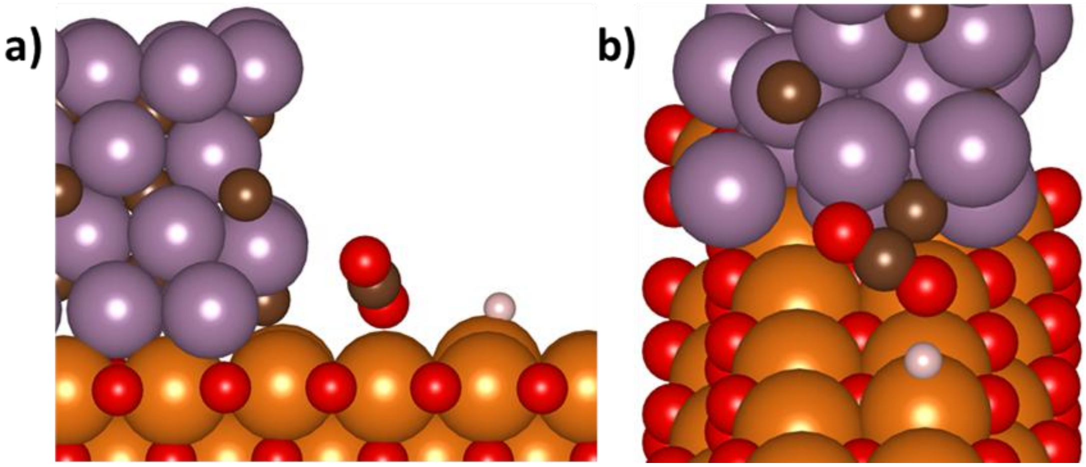

Figure S2. Relaxed images from two different angles, demonstrating that the proximity of an individual H atom to the Mo_2C ribbon is important to activate CO_2.

References

1. Holder, C.F.; Morse, J.R.; Barboun, P.M.; Shabaev, A.R.; Baldwin, J.W.; Willauer, H.D. Evaluating Metal Oxide Support Effects on the RWGS Activity of Mo_2C Catalysts. *Catal. Sci. Technol.* **2023**, *13*, 2685–2695, doi:10.1039/D3CY00026E.
